# I(f) current channel inhibitor (ivabradine) deserves cardioprotective effect via down-regulating the expression of matrix metalloproteinase (MMP)-2 and attenuating apoptosis in diabetic mice

**DOI:** 10.1186/1471-2261-14-150

**Published:** 2014-10-31

**Authors:** Shao-Liang Chen, Zuo-Ying Hu, Guang-Feng Zuo, Ming-Hui Li, Bin Li

**Affiliations:** Department of Cardiology, Nanjing First Hospital, Nanjing Medical University, 68 Changle Road, Nanjing, 210006 China; Department of Cardiology, Nanjing Heart Center, Nanjing, China; Third Clinical Medical College, Nanjing Medical University, Nanjing, China; Nanjing Medical University, Nanjing, China

**Keywords:** Diabetes, Gene, Microarray, Signal pathway, Immunohistochemistry, Apoptosis

## Abstract

**Background:**

Ivabradine (IVBD), a novel I(f)-channel inhibitor and specific heart rate-lowering agent, is known to have anti-oxidative activity that promotes endothelial function. However, the molecular mechanism through which IVBD acts on cardiac function has yet to be elucidated, especially in experimental diabetic animals.

**Methods:**

For this reason, twenty diabetic mice were randomly assigned to IVBD-treated (10 mg/kg/day) and control (saline) groups. After a 3-month treatment, microarray assay was performed to identify differentia expressed genes, and cardiac function was measured by echocardiography, with subsequent immunohistochemistry analysis and western blotting.

**Results:**

Our results showed that ivabradine treatment attenuated the expression and staining score of matrix metalloproteinase (MMP)-2, induced the dephosphorylation of caspase 3, BAX and MMP-2, and enhanced the phosphorylation of NF-κB. Ivabradine treatment led to a significant improvement in cardiac function.

**Conclusion:**

Ivabradine significantly improved cardiac function by attenuating apoptosis and inhibiting the expression and activity of MMP-2 in diabetic mice, which underscored the novel clinical implications of ivabradine for diabetic patients.

## Background

In 2007, roughly 6% of people were affected worldwide by diabetes and it is estimated that this will increase to 7.3% by 2025 [1]. Diabetes, 90% of which is type 2 diabetes (T2D), is characterized by impaired glucose homeostasis and decreased insulin activity and insulin resistance [2], which lead to elevated blood glucose levels and multiple system complications [3]. Endothelial dysfunction in T2D leads to obstructive coronary arterial stenosis, which could be partially prevented and minimized by the administration of beta-blockers because of their heart rate (HR)-lowering effect [4]. However, severe side effects from beta-blockers, including reduced heart function and blood pressure, limit its use in certain patients.

Ivabradine (IVBD), a novel If-channel inhibitor and specific HR-lowering agent, acts on the sinoatrial node but does not alter ventricular contractility and vascular tone, and has been used for certain patients with angina pectoris or heart failure who are intolerable to beta-blockers [5]. Theoretically, IVBD treatment should be beneficial in T2D, unfortunately, this has not been tested in diabetic state.

Furthermore, previous studies about the mechanisms and effect of IVBD on endothelial protection in non-diabetic animals or cells introduced conflict results [6–9], as such favoring eNOS expression and/or the prevention of NO or H_2_O_2_ degradation [6], inhibiting NADPH-oxidase activity, superoxide release and the renin-angiotensin-aldosterone system (RAAS) in ApoE^-/-^ mice [7] ,and not up-regulating the aortic PI3K/Akt/eNOS signaling system [8] which is different to the finding by Walcher et al. [9] who reported that IVBD inhibits the chemokine-induced migration of CD4-positive lymphocytes by limiting both PI-3 kinase activity and the phosphorylation of AKT.

Notably, the biological and molecular effects ofIVBD on the heart in the diabetic state has not been studied previously. Accordingly, the present study aims to determine the improvement in cardiac function after administering IVBD to diabetic mice and to explore the mechanisms by which IVBD acts on.

## Methods

### Preparation of animals

All diabetic mice were male mices purchased from Nanjing Experimental Center (Nanjing, China) when they were 11–12 weeks old and were housed in a temperature and humidity controlled animal facility with a 12:12 hr light–dark cycle. All rats were fed Teklad Global 18% Protein Rodent Diet (Harlan Laboratories Asia, Hongkong, China) and had free access to water. All protocols in this study were conducted in accordance with the National Health guidelines and were approved by the Institutional Animal Care and Use Committee of Nanjing Medical University (Nanjing, China).

### Grouping of animals

Twenty diabetic mice were randomly divided into two groups (of 10 rats each): an IVBD-10 group (ivabradine was added in the diet at a dosage of 10 mg/kg/day for three months), and a control group (ivabradine was replaced with saline, which was administered for 3 months).

### Measurement of cardiac function by echocardiography

Before and after 3 months of IVBD or saline administration, transthoracic echocardiography (TTE) was performed (MyLab25, Esaote, Italy) with a 13 MHz linear array transducer. Under light anesthesia (ketamine HCl and xylazine, 75 and 3.5 mg/kg body weight, respectively, delivered intraperitoneally), the left ventricular (LV) wall thickness and end-diastolic (LVDd) and end-systolic diameters (LVDs) were determined from the short-axis view at the midpapillary level. The total LV mass (LVM) and corrected LVM were calculated. The LV end-diastolic and end-systolic volumes were planimetered from the parasternal long-axis view. The LV ejection fraction (LVEF) was calculated as (LV diastolic volume – LV systolic volume)/LV diastolic volume. All echocardiographic investigations were performed according to the recommendations of the American Society of Echocardiography [10]. In the current study, the intra- and inter-observer error(s) were calculated according to s = SD/√2. The intra-observer variability for the measurement of LVEF was <5%.

### Tissue samples

At 3 months, 5 animals in each group were sacrificed by pentobarbital overdose. The hearts were harvested and rinsed in PBS. Homogenize tissue samples in 1 ml of TRIZOL Reagent per 50–100 mg of tissue using a power homogenizer. The sample volume should not exceed 10% of the volume of TRIZOL Reagent used for homogenization. The cells were then washed and incubated in SmBM + 0.5% FBS. The reactions were performed in quadruplicates.

### Cells grown in monolayer

Lyse cells directly in a culture dish by adding 1 ml of TRIZOL Reagent to a 3.5 cm diameter dish, and passing the cell lysate several times through a pipette. The amount of TRIZOL Reagent added is based on the area of the culture dish (1 ml per 10 cm2) and not on the number of cells present.

### Cells grown in suspension

Pellet cells by centrifugation. Lyse cells in TRIZOL Reagent by repetitive pipetting. Use 1 ml of the reagent per 5–10 × 10^6^ of animal, plant or yeast cells, or per 1 × 10^7^ bacterial cells. Washing cells before addition of TRIZOL Reagent should be avoided as this increases the possibility of mRNA degradation. Disruption of some yeast and bacterial cells may require the use of a homogenizer.

### Phase separation

Incubate the homogenized samples for 5 minutes at 15 to 30°C to permit the complete dissociation of nucleoprotein complexes. Add 0.2 ml of chloroform per 1 ml of TRIZOL Reagent. Cap sample tubes securely. Shake tubes vigorously by hand for 15 seconds and incubate them at 15 to 30°C for 2 to 3 minutes. Centrifuge the samples at 12,000 × *g* for 15 minutes at 4°C. Following centrifugation, the mixture separates into a lower red, phenol-chloroform phase, an interphase, and a colorless upper aqueous phase. RNA remains exclusively in the aqueous phase. The volume of the aqueous phase is about 60% of the volume of TRIZOL Reagent used for homogenization.

### Rna precipitation

Transfer the aqueous phase to a fresh tube. Precipitate the RNA from the aqueous phase by mixing with isopropyl alcohol. Use 0.5 ml of isopropyl alcohol per 1 ml of TRIZOL Reagent used for the initial homogenization. Incu4bate samples at 15 to 30°C for 10 minutes and centrifuge at 12,000 × *g* for 10 minutes at 4°C. The RNA precipitate, often invisible before centrifugation, forms a gel-like pellet on the side and bottom of the tube.

### Rna wash

Remove the supernatant. Wash the RNA pellet once with 75% ethanol, adding at least 1 ml of 75% ethanol per 1 ml of TRIZOL Reagent used for the initial homogenization. Mix the sample by vortexing and centrifuge at 7,500 × *g* for 5 minutes at 4°C.

### Redissolving the rna

At the end of the procedure, air-dry the RNA pellet for 5–10 minutes. Do not dry the RNA by centrifugation under vacuum. It is important not to let the RNA pellet dry completely as this will greatly decrease its solubility. Partially dissolved RNA samples have an A260/280 ratio <1.6. Dissolve RNA in RNase-free water by passing the solution a few times through a pipette tip, and incubating for 10 minutes at 55 to 60°C. RNA can be stored at -70°C.

### Isolation of small quantity rna

Isolation of RNA from small quantities of tissue (1 to 10 mg) or Cell (10^2^ to 10^4^) Samples: Add 800 μl of TRIZOL to the tissue or cells. Following sample lysis, add chloroform and proceed with the phase separation as described in step 2. Prior to precipitating the RNA with isopropyl alcohol, add 5-10 g RNase-free glycogen as carrier to the aqueous phase. To reduce viscosity, shear the genomic DNA with 2 passes through a 26 gauge needle prior to chloroform addition. The glycogen remains in the aqueous phase and is co-precipitated with the RNA. It does not inhibit first-strand synthesis at concentrations up to 4 mg/ml and does not inhibit PCR.

### Denaturing agarose gel electrophoresis

Heat 1 g agarose in 72 ml water until dissolved, then cool to 60°C. Add 10 ml 10X MOPS running buffer, and 18 ml 37% formaldehyde (12.3 M). Pour the gel and allow it to set. The wells should be large enough to accommodate at least 25 μl. Remove the comb, and place the gel in the gel tank. Add enough 1X MOPS running buffer to cover the gel by a few millimeters. To 3 μg RNA, add 3X volumes Formaldehyde Load Dye. Ethidium bromide can be added to the Formaldehyde Load Dye at a final concentration of 10 μg/ml. Heat denature samples at 65–70°C for 15 min.

Load the gel and electrophorese at 5–6 V/cm until the bromophenol blue (the faster-migrating dye) has migrated at least 2–3 cm into the gel.

**Figure 1:**
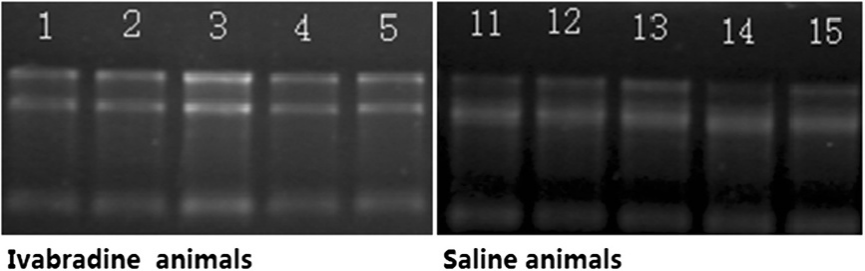
**RNA denaturing agarose gel electrophoresis for ivabradine and control groups.** Animals in the ivabradine group were numbered as 1–5 and as 11–15.

The 28S and 18S ribosomal RNA bands should be fairly sharp, intense bands (size is dependent on the organism from which the RNA was obtained). The intensity of the upper band should be about twice that of the lower band. Smaller, more diffuse bands representing low molecular weight RNAs (tRNA and 5S ribosomal RNA) may be present. It is normal to see a diffuse smear of ethidium bromide staining material migrating between the 18S and 28S ribosomal bands, probably comprised of mRNA and other heterogeneous RNA species. DNA contamination of the RNA preparation (if present) will be evident as a high molecular weight smear or band migrating above the 28S ribosomal RNA band. Degradation of the RNA will be reflected by smearing of ribosomal RNA bands (Table 1, Figure 1).

**Table 1:** **RNA yield and quality before and after DNase I treatment**

Samples before Dnase I treatment	OD _260_	OD _280_	OD _260_/ OD _280_	RNA concentration(ng/μl)
IVBD-1	17.079	8.765	1.95	683.14
IVBD-2	15.494	7.962	1.95	619.77
IVBD-3	5.603	3.829	1.46	224.11
IVBD-4	11.815	5.889	2.01	472.58
IVBD-5	17.626	8.540	2.06	705.05
Control-1	13.908	7.215	1.93	556.32
Control-2	9.294	4.841	1.92	371.75
Control-3	11.153	5.965	1.87	446.14
Control-4	14.234	7.344	1.94	569.36
Control-5	14.797	7.684	1.93	591.89
**Samples after DNase I treatment**	**OD**_**260**_	**OD**_**280**_	**OD**_**260**_ **/ OD**_**280**_	**RNA concentration(ng/ul)**
IVBD-1	7.994	3.939	2.03	319.76
IVBD-2	6.840	3.493	1.96	273.59
IVBD-3	5.417	2.769	1.96	216.68
IVBD-4	8.579	4.371	1.96	343.16
IVBD-5	12.352	6.073	2.03	494.08
Control-1	4.455	2.307	1.93	178.20
Control-2	4.807	2.405	2.00	192.27
Control-3	10.141	5.244	1.93	405.63
Control-4	10.424	5.408	1.93	416.96

IVBD, ivabradine.

### Real-time polymerase chain reaction (RT-PCR) analysis

The extracted RNA was first DNase-treated with RQ1 RNase-Free DNase (Promega, USA) and heat inactivated according to the manufacturer’s protocol. The threshold cycle (CT) value for amplification of each gene was determined by the auto threshold function of the software. Prior to the amplification, the PCR efficiency and primers compatibility for the gene of interest and a reference gene were validated via the standard curve method [10]. A melting curve analysis with temperature ramping from 55°C–99°C was carried out for each run to confirm the specificity of the PCR amplifications. *β-*actin*,* which served as a reference gene, was used for the normalization of the cDNA input. Actb, B2M and Gusb served as housekeeping (HK) genes.

### Microarray analysis

Calculate the ΔCt for each pathway-focused gene in each treatment group [11, 12]: ΔCt (group 1) = average Ct – average of HK genes’ Ct for group 1 array; ΔCt (group 2) = average Ct – average of HK genes’ Ct for group 2 array. Calculate the ΔΔCt for each gene across two PCR Arrays (or groups): ΔΔCt = ΔCt (group 2) - ΔCt (group 1), where group 1 is the control and group 2 is the experimental. Finally, the fold-change for each gene from group 1 to group 2 as 2^-ΔΔCt^ was calculated.

### Immunohistochemical study

Animals hearts were fixed in 4% paraformaldehyde for 24 h, embedded in paraffin, and cross-sectioned into 10-mm slices. Sections were stained with hematoxylin and eosin (H&E) for cell alignment according to the manufacturer’s instructions. Based on the results of the microarray analysis, staining was performed to determine the expression of MMP-2, TGF-β, TIMP and P53. The staining score was calculated based on the percent positive area (no positive staining = 0; less than 25% = 1 point; 25–50% = 2 points; 51–75% = 3 points; and more than 75% = 4 points) multiplied by the staining intensity (weak = 1; moderate = 2; strong = 3, and very strong = 4). Five fields of view were randomly selected for each sample, and the average positive points for each case were reported as the actual positive points.

### Western blot analysis

Cardiomyocytes were gently washed twice with ice-cold PBS and lysed in a cocktail of RIPA buffer, proteinase inhibitor and phosphatase inhibitor. After 20 min on ice, cells were scraped and lysates were clarified by centrifugation at 12,000 x g for 15 min at 4°C. Protein concentrations were quantified by BCA protein assay according to the manufacturer’s instructions. In total, 60 μg of protein were separated by a 10% SDS-PAGE gel and transferred to a PVDF membrane before being incubated overnight at 4°C with a primary antibody (caspase 3, BAX, NF-κB, MMP-2 and β-actin). After that the membrane was washed and incubated with a horseradish peroxidase-conjugated secondary antibody for 1 hour at room temperature. After a second wash, the membranes were developed using an enhanced chemiluminescence substrate and the band intensities were analyzed using ImageJ (National Institutes of Health, Bethesda, USA).

### Statistical analysis

Data are presented as mean ± SD for continuous variables and frequency for categorical variables. Comparison of continuous variables was performed using the *t* test or Mann–Whitney test as appropriate. The chi-square test or Fisher's exact test was used to analyze categorical variables. All statistical analyses were performed using SPSS® software, version 17.0 (SPSS Inc., USA). Statistical significance was set as *P* < 0.05.

## Results

### Measurement of cardiac function by echocardiography

**Figure 2:**
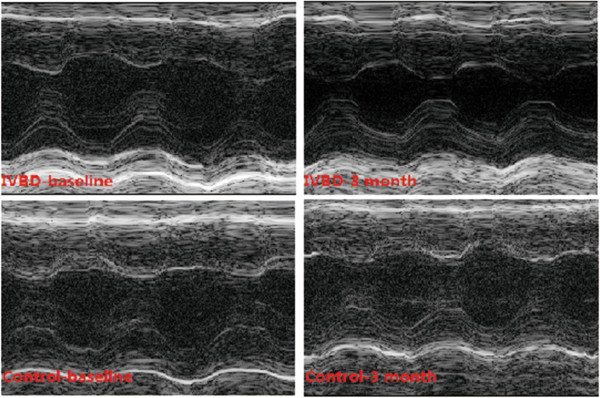
**Comparison of cardiac function between ivabradine and control groups.** M-model echocardiography showed a significant increase of ventricular wall movement after ivabradine treatment (upper column); however, there was no significant difference in cardiac function in the control group (lower column).

Indices of cardiac function were comparable between the ivabradine and control groups (Table 2 and Figure 2). Importantly, at the 3 month follow-up, IVSTs, LVDs, LVPWs, eject fraction (EF) and fractional shortening (FS) improved significantly in the ivabradine group (1.59 ± 0.03 mm, 1.65 ± 0.08 mm, 1.42 ± 0.09 mm, 75.7 ± 3.43% and 49.33 ± 3.01 %, respectively) compared with the control group (1.41 ± 0.21 mm, p = 0.022; 2.22 ± 0.53 mm, p = 0.017; 1.26 ± 0.14 mm, p = 0.032; 54.1 ± 8.3 %, p = 0.018; and 27.34 ± 4.87%, p < 0.001, respectively). Compared with the baseline measurements, at 3 months after administration of ivabradine, cardiac function was significantly improved with the exception of the left ventricular volume at end-diastole. However, there were no significant differences in echocardiographic measurements between the baseline and the 3 month follow-up in the control group.

**Table 2:** **Measurements of cardiac function by echocardiography**

	Ivabradine (n = 7)	Control(n = 6)	P value
**IVSTd (mm)**			
Baseline	0.95 ± 0.12	0.89 ± 0.18	0.539
At 3 months	1.09 ± 0.12	0.94 ± 0.15	0.089
**IVSTs (mm)**			
Baseline	1.37 ± 0.17	1.29 ± 0.21	0.468
At 3-month	1.59 ± 0.03	1.24 ± 0.21	0.022
**LVDd (mm)**			
Baseline	3.30 ± 0.31	3.33 ± 0.27	0.932
At 3-month	2.99 ± 0.14	3.24 ± 0.25	0.497
**LVDs (mm)**			
Baseline	2.09 ± 0.17	2.26 ± 0.50	0.436
At 3-month	1.65 ± 0.08	2.22 ± 0.53	0.017
**LVPWd (mm)**			
Baseline	0.92 ± 0.18	0.84 ± 0.12	0.353
At 3-month	1.09 ± 0.09	0.94 ± 0.16	0.067
**LVPWs (mm)**			
Baseline	1.21 ± 0.09	1.18 ± 0.11	0.577
At 3-month	1.42 ± 0.09	1.26 ± 0.14	0.032
**EF (%)**			
Baseline	67.1 ± 6.96	61.6 ± 7.6	0.202
At 3-month	75.7 ± 3.43	54.1 ± 8.3	0.018
**FS (%)**			
Baseline	36.42 ± 5.56	31.66 ± 4.96	0.135
At 3-month	49.33 ± 3.01	27.34 ± 4.87	<0.001
**LVM (g)**			
Baseline	107.55 ± 10.75	99.81 ± 15.88	0.596
At 3-month	85.85 ± 13.04	98.39 ± 27.92	0.309
**LVM-corrected(mm)**			
Baseline	86.04 ± 8.59	79.99 ± 28.77	0.605
At 3-month	66.65 ± 10.51	78.60 ± 22.30	0.230
**LVDd (ml)**			
Baseline	44.84 ± 10.25	51.99 ± 25.65	0.509
At 3-month	48.07 ± 6.69	49.32 ± 28.40	0.911
**LVSd (ml)**			
Baseline	14.42 ± 2.67	19.79 ± 10.24	0.206
At 3-month	11.97 ± 2.02	19.82 ± 11.06	0.091

**IVST,** inter-ventricular septal thickness; **d,** end-diastolic; **s**, end-systolic; **LVD**, left ventricular dimension; **LVPW**, left ventricular posterior wall; **EF**, eject fraction; **FS**, fraction of shortness; **LVM**, left ventricular mass.

### Differentially expressed genes in the myocardium of diabetic mice

According to microarray analysis, 15 genes were differentially expressed in diabetic mice after ivabradine treatment. Of these 15 genes, MMP-2 was down-regulated (fold-change, defined as the ratio of control/ivabradine, 2.459, p = 0.031), and the remaining 14 genes were up-regulated (Table 3).

**Table 3:** **Genes with significantly higher expression (>2 times) in the ivabradine group than in the control group**

Gene name	Function	IVBD (mean)	Control (mean)	FC	P
MMP -2(matrix metallopeptidase- 2)	ECM	0.027	0.067	0.40	0.031
MIP-3 β(macrophage inflammation protein-3 β, CCL 19)	Lymphoid T cells	0.319	0.144	2.22	0.014
6Ckine(chemokine with 6 cysteines,CCL21)	Lymphoid organs	0.421	0.177	2.37	0.024
ACE/CD143(angiogensin I Converting Enzyme/CD 143)	RAAS	0.433	0.189	2.29	0.006
ALK-1(activin receptor-like kinase)	TGF- β	0.327	0.123	2.66	0.047
CT-1(cardiotrophin-1)	apoptosis	0.292	0.086	3.39	0.016
CD27(CD-27)	apoptosis	0.245	0.089	2.75	0.021
CD27 Ligand(CD70)	TNF	0.212	0.067	3.16	0.020
Endoglin(endoglin)	TGF- β	0.333	0.159	2.09	0.005
Epigen(epigen)	EGF-like	0.173	0.0845	2.05	0.039
IL-17E(interlukin-17E)	T cells	0.327	0.161	2.03	0.001
IL-17 F(interlukin-17 F)	T cells	0.385	0.163	2.36	0.001
IL-1ra/IL-1 F3(interlukin-1ra/-1 F3)	T cells	0.266	0.121	2.19	0.003
IL-2 Rα(interlukin-2 Rα)	T cells	0.298	0.143	2.08	0.001

Fold changes are relative to normal samples.

**FC**, fold change; **ECM**, extracellular matrix; **RAAS**, renin angiotensin aldosterone system; **TGF**, tissue growth factor; **TNF**, tumor necrosis factor; **EGF**, epidermal growth factor.

### Functional gene sets that discriminate between ivabradine and control groups

From these significant diabetic development genes, we have been able to identify some specific functional groups. MMP-2 is one member of the matrix metalloproteinase family, which modulates the inflammatory system and apoptosis. MMP-2 targeting at the extracellular matrix was significantly inhibited by ivabradine. Lymphocyte proliferation induced by inflammation and immunity was markedly promoted by ivabradine, and this was confirmed by the significant up-regulation of 6Ckine, ACE/CD143, ALK-1, CT-1, CD27, endoglin, MIP-3β, epigen, IL-17E/F, IL-1ra/IL-1 F3, and IL-2 Rα.

### Immunohistochemical study

**Figure 3:**
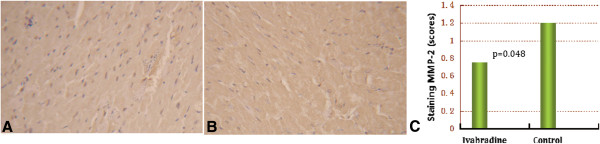
**Comparison of MMP-2 staining before (A) and 3 months after (B) ivabradine treatment.** The difference in staining score reached significance (p = 0.048) **(C)**.

Based on the microarray analysis, the up-regulation of MMP-2 expression was analyzed by immunostaining, and the results are shown in Table 4 and Figure 3. The mean staining score and staining intensity in the ivabradine group were significantly improved (2.1 ± 0.2 scores and 1.9 ± 0.3 grade), compared with the control group (3.6 ± 0.3 scores, p = 0.038 and 3.9 ± 0.3 grade, p = 0.022, respectively).

**Table 4:** **Immunohistochemistry analysis**

	ivabradine (n = 6)	Control (n = 6)	P value
**Matrix metalloproteinase-2**			
Staining score			
Median (75% quartile)*	0.75 (1.00)	1.5 0(1.25)	0.048
Mean (95%CI)	0.75 (0.29-0.92)	1.2 (0.64-1.76)	0.032
Staining intensity, grade	1.9 ± 0.3	3.9 ± 0.3	0.022
Week + moderate, n(%)	4 (66)	1 (17)	0.043
Strong + very strong, n(%)	2 (14)	5 (83)	0.043

*Non-normal data distribution. Nonparametric analysis was used for the comparison between the two groups.

### Western blot analysis

**Figure 4:**
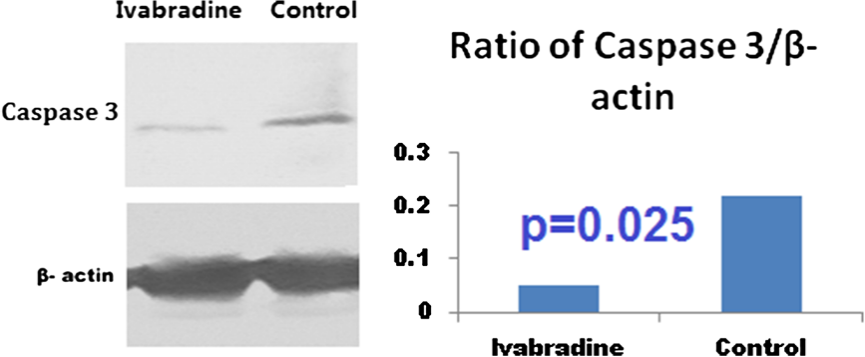
**Western blot analysis of the phosphorylation state of caspase 3.** Caspase 3 was dephosphorylated significantly in the ivabradine group, compared with the control group.

**Figure 5:**
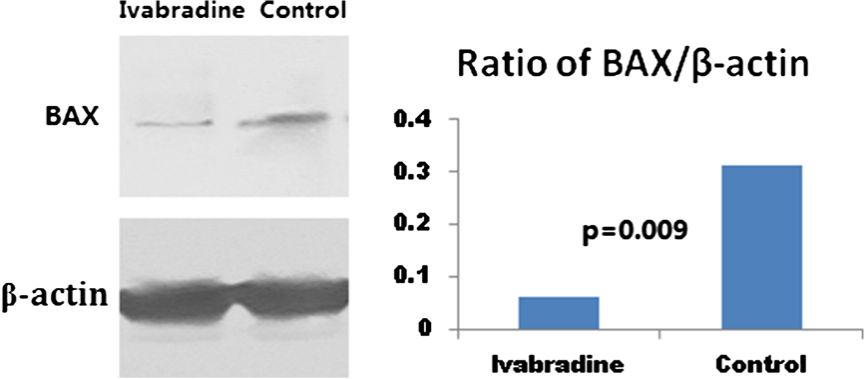
**Western blot analysis of the phosphorylation of BAX.** BAX was dephosphorylated significantly in the ivabradine group, compared with the control group.

**Figure 6:**
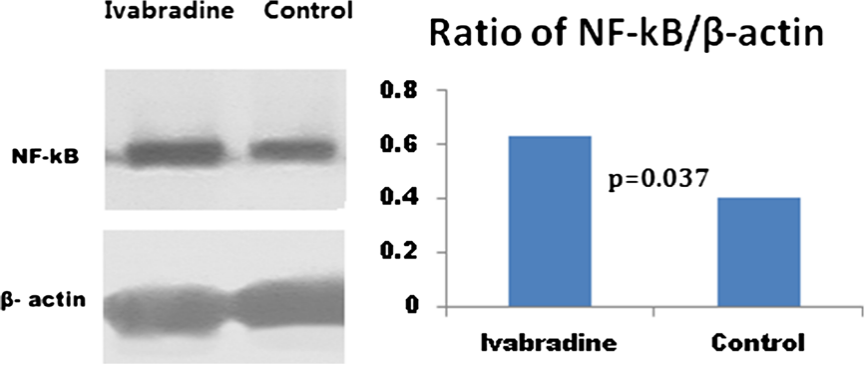
**Western blot analysis of the phosphorylation of NF-κB.** Phosphorylation of NF-κB was increased significantly in the ivabradine group, compared with the control group.

**Figure 7:**
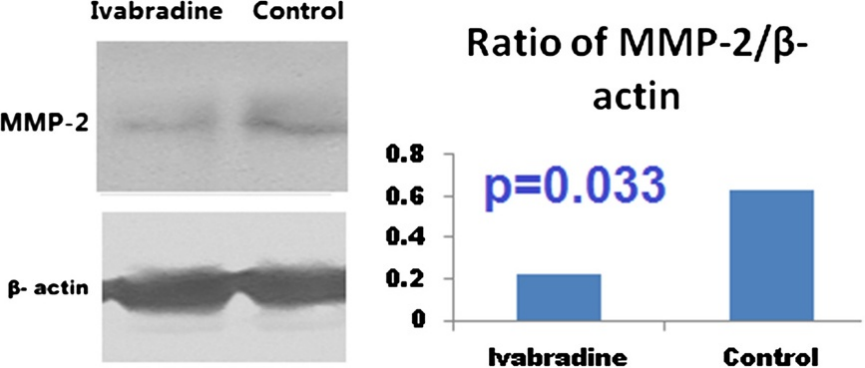
**Western blot analysis of the phosphorylation of MMP-2.** MMP-2 was dephosphorylated significantly in the ivabradine group, compared with the control group.

Based on the results of the microarray, we investigated the phosphorylation of MMP-2 and several other proteins involved in apoptosis, including NF-κB, caspase 3 and BAX. Phosphorylation of caspase 3 and BAX was significantly reduced by ivabradine (Figure 4 and Figure 5), consistent with the increased phosphorylation of NF-κB after treatment by ivabradine (Figure 6). In line with the microarray analysis, MMP-2 phosphorylation was inhibited in ivabradine-treated rats (Figure 7) compared with the control group.

## Discussion

The major findings of the present study are: (1) Ivabradine treatment significantly inhibits the expression and activity of MMP-2 in diabetic mice, (2) Ivabradine down-regulates the phosphorylation of Caspase 3, BAX, but up-regulats the phosphorylation of NF-kB in mice with diabetes. Taken together, heart function of diabetic nimals is improved significantly during Ivabradine treatment, compared to control group.

A number of genes have been discovered as potential candidates to cause T2D via the modulation of different signal pathways mediated [13–24]. Cross-talk between these signaling systems unravels the complexity of the molecular mechanism of T2D.

Matrix metalloproteinases (MMPs) are a family of zinc-binding proteolytic enzymes that normally remodel the extracellular matrix and pathologically attack substrates as part of an inflammatory response. The major MMP species in the myocardium and vasculature are the gelatinases (MMPs 2 and 9), MMP-1 (interstitial collagenase) and Mt1-MMP. Recently it has been proven that matrix metalloproteinases (MMPs) play an important role in atherosclerosis and the rebuilding of the vascular wall [25]. The alteration of MMP-2 gene expression induced by a 3 month ivabradine treatment, was associated with an improvement of cardiac function in diabetic mices, as confirmed by our immunohistochemistry and Western blot analyses.

It has been found that high glucose concentration promotes TGF-β expression and activates the Jak/STAT signaling cascade in diabetic kidney cells. Activation of this signaling cascade can stimulate excessive proliferation and the growth of glomerular mesangial cells, contributing to diabetic nephropathy [14, 15]. Exposure to high glucose concentrations has also been shown to activate the MAPK signaling pathway in skeletal muscle cells [16]. These findings were in agreement with our finding that ivabradine improved cardiac function by promoting the expression and activity of endoglin. Endoglin is an auxiliary receptor for the TGF-β receptor complex, which functions in related signaling pathways and is mainly expressed in vascular and connective tissues and in endothelial and stromal cells [26, 27]. Up-regulated endoglin expression has been reported during wound healing and tumor vascularization, and in inflammatory tissues and developing embryos [26–28]. Mutations in endoglin have been found to be a causal factor in hereditary hemorrhagic telangiectasia (HHT), a disease characterized by the malformation of vascular structure [29, 30]. We postulated that the up-regulation of endoglin expression by ivabradine would be related to the impairment of malformed vascular structure [29–31].

PI3K/Akt is a key molecule in insulin signaling that is found to be down-regulated in T2D [20]. However, there is a discrepancy in terms of the regulation of the PI3K/Akt transduction pathway by ivabradine: up-regulating eNOS expression independent of PI3K/Akt pathway [5], inhibiting NADPH/ROS/RAAS but regulating PI3K/Akt in ApoE^-/-^ mice [6, 7], limiting PI3K activity and the phosphorylation of AKT in CD4-positive lymphocytes [8]. We found that the expression and activity of the epigen gene was up-regulated by ivabradine treatment in diabetic myocardium. Epigen encodes a protein of 152 amino acids that contains EGF-like features. Epigen exhibits 24–37% sequence identity with EGF, TGF, and epiregulin. EGF exerts insulin-like effects on glucose transport and lipolysis and can increase the tyrosine phosphorylation and activation of IRS-1 and IRS-2. EGF is also capable of activating additional PI3K pools, thereby augmenting the downstream signaling of insulin in insulin-resistant states like T2D [13]. As a result, the modulation of epigen expression and activity by ivabradine via PI3K and MAPK signaling [32] would be predicted to be associated with the improvement of cardiac function. Similarly, MIP 3-β also regulated both the P38/MAPK and PI3K/Akt signaling pathways [33–35]. However, there was a lack of direct evidence of ivabradine regulating the PI3K/Akt signaling system provided by our results, as the change of Akt expression and Combo protein were not significantly different between the ivabradine and control groups.

Caspase 3 and BAX are two signals participating in the process of apoptosis [35, 36]. Our study showed that ivabradine was associated with the significant dephosphorylation of caspase 3 and BAX, indicating the anti-apoptotic effect of ivabradine in diabetic animals. In contrast, NF-κB was activated by ivabradine, and phosphorylated NF-κB would promote protein synthesis and thus inhibit apoptosis [37]. These results would provide additional evidences of anti-ischemic effect by ivabradine for diabetic setting, in line with previously published results confirming the cardioprotective effect by ivabradine for patients with ischemic heart disease [38, 39].

### Limitation

Obviously, the current study did not analyze the mechanism attributive to the signal pathways in which ivabradine involved. For example, the question why NF-kB was activated by ivabradine was not studied. Another limitation was small sample size, which would be expanded in our next study. Finally, we did not performed Western Blotting analysis for all differentially expressed genes, which would be at least mask the potential of cross-talking by different signal pathways.

## Conclusion

Our study for the time reported the cardioprotective effect by ivabradine in diabetic animal. The major finding would be implied the possible benefits of ivabradine for diabetic patients. As a result, further clinical study is required in order to elucidate the efficacy and safety of ivabradine for Type 2 diabetes.
